# Diffusion Tensor Imaging Versus Conventional MRI in Temporal Lobe Epilepsy: A Comparative Study of Diagnostic Performance

**DOI:** 10.7759/cureus.96902

**Published:** 2025-11-15

**Authors:** Saptarshi Roy, Manish Dagar, A Baskar, Shraddha Sharma, Piyoosh Priyadarshee

**Affiliations:** 1 Radiology, Sree Balaji Medical College and Hospital, Chennai, IND; 2 Radiodiagnosis, Sree Balaji Medical College and Hospital, Chennai, IND

**Keywords:** apparent diffusion coefficient, diffusion tensor imaging, fractional anisotrophy, temporal lobe epilepsy, white matter integrity

## Abstract

Background: Conventional magnetic resonance imaging (MRI) may appear normal in a proportion of patients with temporal lobe epilepsy (TLE). Diffusion tensor imaging (DTI) provides quantitative measures of white matter microstructure and may reveal subtle changes not visible on routine MRI. This study compared DTI-derived diffusion metrics with standard MRI epilepsy protocols in patients with unilateral TLE to assess the extent and pattern of white matter alterations.

Methods: A cross-sectional observational study was conducted in the Department of Radiodiagnosis at Sree Balaji Medical College and Hospital, Chennai, Tamil Nadu, India, with all MRI and DTI studies performed on a GE Signa Pioneer 3.0T system (GE Healthcare, DV26.0 software) using a dedicated 32-channel head coil. Twenty-one patients with confirmed TLE and 11 age-matched controls underwent both conventional MRI and DTI. Regions of interest (ROIs) were placed on the hippocampus, fornix, thalamus, corpus callosum, internal capsule, cingulum, and inferior fronto-occipital fasciculus (IFO) tracts. Fractional anisotropy (FA) and apparent diffusion coefficient (ADC) values were analyzed using IBM SPSS Statistics version 26.

Results: DTI revealed statistically significant alterations in FA and ADC values across multiple brain regions, including the hippocampus (FA: p < 0.05, ADC: p < 0.05), thalamus (FA: p << 0.001, ADC: p << 0.001), corpus callosum (FA: p << 0.001-0.05), internal capsule (FA: p << 0.001-0.05), and IFO tracts (FA and ADC: p << 0.001), demonstrating higher diagnostics compared to conventional MRI.

Conclusions: This study highlights that DTI offers additional microstructural information beyond conventional MRI in unilateral TLE. DTI may therefore serve as a complementary imaging tool, particularly in cases where standard MRI appears normal. Further diagnostic accuracy studies are required before DTI can be considered a superior or standalone modality.

## Introduction

Epilepsy is one of the most common neurological disorders worldwide, characterized by recurrent, unprovoked seizures resulting from abnormal neuronal discharges. Among the imaging techniques available for its diagnosis and management, magnetic resonance imaging (MRI) plays a pivotal role due to its superior soft-tissue contrast and multiplanar capability. Despite the high diagnostic yield of conventional MRI, approximately 20-30% of patients with focal epilepsy exhibit no visible abnormalities on standard scans, classifying them as “MRI-negative” cases. This limitation suggests that conventional MRI techniques may lack sufficient sensitivity to detect subtle or microstructural alterations underlying epileptogenic zones. Ongoing advances in magnetic field strengths, receiver coil technology, and post-processing algorithms have significantly enhanced the resolution and diagnostic capabilities of MRI; however, the development of novel contrast agents has not yet achieved comparable improvements in lesion detectability [1,2].

Temporal lobe epilepsy (TLE) is one of the most prevalent and complex forms of focal epilepsy, accounting for a substantial proportion of all cases. It is characterized by recurrent seizures originating from the temporal lobes and is frequently associated with structural abnormalities such as mesial temporal sclerosis (MTS) or hippocampal atrophy. Clinically, TLE is often refractory to antiepileptic drugs, rendering it one of the most challenging epilepsies to manage. In patients with medically refractory TLE, surgical resection of the epileptogenic zone, most commonly via anterior temporal lobectomy, can offer significant seizure reduction or even complete remission. Therefore, accurate imaging is indispensable for diagnosis, presurgical localization, and postoperative follow-up. MRI is central to these processes, as it enables both the visualization of structural lesions and the evaluation of subtle pathological alterations that guide surgical decision-making [3-5].

Conventional MRI protocols for epilepsy employ high-resolution T1- and T2-weighted sequences to identify structural lesions such as MTS, cortical dysplasias, or tumors that may act as epileptogenic foci. High-resolution coronal T2-weighted images perpendicular to the hippocampal long axis are particularly useful in detecting hippocampal atrophy and signal alterations indicative of neuronal loss and gliosis. Quantitative MRI techniques, including hippocampal volumetry and T2 relaxometry, have further improved sensitivity in identifying subtle hippocampal pathology. However, despite these technical refinements, up to one-third of patients with focal epilepsy continue to demonstrate normal findings on conventional MRI, underscoring the need for more advanced imaging techniques capable of revealing microstructural abnormalities invisible on standard scans [6-8].

Diffusion-weighted imaging (DWI) and its more sophisticated extension, diffusion tensor imaging (DTI), have emerged as promising tools to overcome these limitations. DTI provides insights into the microstructural organization of brain tissue by quantifying the diffusion of water molecules, a process that reflects underlying tissue architecture. While conventional MRI primarily detects macroscopic structural changes, DTI measures the anisotropy and magnitude of water diffusion in multiple directions, thus revealing information about axonal integrity, fiber orientation, and connectivity. By applying tensor modeling to the diffusion data, DTI generates detailed maps of the brain’s white matter tracts and allows the computation of quantitative diffusion metrics [9].

Among the principal DTI-derived parameters, fractional anisotropy (FA) reflects the degree of directionality of water diffusion and serves as a marker of white matter tract coherence and integrity. Mean diffusivity (MD) measures the overall magnitude of water diffusion, providing an index of cellular density and tissue integrity. Additional parameters such as axial diffusivity (AD) and radial diffusivity (RD) can offer further insights into specific pathological processes - axonal degeneration, demyelination, or gliosis. Alterations in FA and MD have been consistently associated with microstructural abnormalities in epilepsy, rendering DTI a powerful technique for detecting subtle changes in both lesional and non-lesional areas [10].

In patients with TLE, DTI has revealed microstructural abnormalities extending beyond the temporal lobe, suggesting that epilepsy is not merely a focal disorder but involves distributed brain networks. For instance, while conventional MRI typically demonstrates unilateral hippocampal atrophy or sclerosis in mesial temporal lobe sclerosis (MTLS), DTI studies have identified bilateral changes in FA and MD, implicating the contralateral hippocampus and extratemporal regions. These findings reflect the widespread network disruptions caused by epileptic activity and offer a more comprehensive understanding of TLE pathophysiology. Such evidence supports the notion that epilepsy-related microstructural changes may precede or exist independently of overt atrophy detectable on conventional imaging [11].

The hippocampus, being central to memory processing and seizure generation in TLE, remains the most critical region of interest in epilepsy imaging. Mesial temporal sclerosis, characterized by neuronal loss, gliosis, and hippocampal atrophy, represents the most frequent pathological substrate of TLE. While high-resolution T2-weighted MRI can identify gross structural changes in the hippocampus, these techniques may not capture microstructural alterations that occur without overt volume loss or signal change. DTI addresses this diagnostic gap by providing additional metrics that quantify white matter integrity and connectivity, thereby complementing the qualitative information derived from conventional MRI [12].

In addition to voxel-based and tract-based analyses, region-of-interest (ROI) approaches are often utilized in DTI studies. In this method, uniform ROIs are placed over color-coded FA maps co-registered with isotropic T1-weighted images, allowing precise measurement of FA and apparent diffusion coefficient (ADC) values in defined brain regions. These quantitative parameters can then be statistically compared between patients and control groups to identify significant microstructural differences. Furthermore, advancements in connectomics have enabled DTI to assess large-scale structural networks, shedding light on how epileptic processes disrupt connectivity across the brain. Such analyses provide valuable insights into the mechanisms underlying seizure propagation and cognitive dysfunction in TLE [13,14].

Despite its advantages, DTI is not without technical challenges. The technique is highly sensitive to motion artifacts, eddy current distortions, and magnetic field inhomogeneities, all of which can compromise image quality and quantitative accuracy. Interpretation of diffusion metrics must also be performed cautiously, as changes in FA or MD can arise from various pathophysiological processes, including inflammation, edema, demyelination, or gliosis, each influencing diffusion properties differently. Continuous developments in acquisition protocols, artifact correction algorithms, and post-processing tools are progressively improving the reliability and reproducibility of DTI measures in both research and clinical contexts [15].

Given these developments, the current study aims to compare the diagnostic performance of DTI with conventional MRI epilepsy protocols in patients with TLE. Specifically, it seeks to determine whether DTI can enhance the detection of hippocampal abnormalities, provide greater insights into the extent of white matter involvement, and correlate these findings with clinical outcomes. By systematically evaluating the strengths and limitations of both modalities, this study aims to clarify the role of DTI as a potential complementary or alternative imaging approach in the evaluation of TLE. Ultimately, these findings may contribute to establishing DTI as a valuable component in standard epilepsy imaging protocols, improving diagnostic accuracy, guiding surgical planning, and advancing the understanding of TLE pathophysiology. This study aimed to compare the diagnostic accuracy of DTI with the normal MRI epilepsy protocol in TLE patients and to establish DTI as the gold-standard MR imaging sequence for epilepsy.

## Materials and methods

Study design and setting

This prospective, cross-sectional observational study was conducted in the Department of Radiodiagnosis at Sree Balaji Medical College and Hospital, Chennai, Tamil Nadu, India, over a period of 18 months, from July 2023 to December 2024. The institution is equipped with a high-field MRI system and advanced imaging infrastructure, enabling both conventional MRI and advanced DTI acquisitions essential for this study. The research was designed in close collaboration with the Departments of Neurology and Neurosurgery, ensuring standardized clinical assessment, radiological imaging, and interpretation protocols. The interdisciplinary coordination allowed accurate selection of participants who fulfilled the diagnostic criteria for TLE, facilitating both clinical and imaging validation of cases.

The study adopted a cross-sectional observational design aimed at comparing two imaging protocols, namely, the conventional MRI epilepsy protocol and DTI, in patients clinically diagnosed with unilateral TLE. This design allowed the evaluation of both qualitative and quantitative parameters from a single imaging session for each participant, thus reducing time-related confounders. The objective was to determine the comparative diagnostic accuracy of these two modalities in detecting hippocampal and white matter changes characteristic of TLE. Including both patients and healthy controls enabled statistical comparison and enhanced the reliability of diagnostic performance metrics. The design reflected real-world clinical practices, where imaging plays a vital role in diagnosis, treatment planning, and pre-surgical evaluation in epilepsy management.

Study population and sample selection

The study population comprised patients aged 10 years and above who presented to the Neurology and Neurosurgery Departments with clinical features suggestive of TLE. Recruitment was conducted through referral following a comprehensive neurological and electroencephalographic (EEG) evaluation. A total of 32 subjects were enrolled, including 21 patients diagnosed with unilateral TLE and 11 age- and sex-matched healthy controls without any history of seizures or neurological illness. The purposive sampling method was chosen to ensure the inclusion of representative cases covering the typical clinical and demographic spectrum of TLE observed in the institution. Recruitment continued until the required sample size, determined by power calculation and feasibility, was achieved.

All enrolled patients underwent thorough clinical evaluation, including seizure history, EEG, and neurological examination, to confirm the diagnosis before imaging. Participants were informed in detail about the study objectives and procedures. Written informed consent was obtained from all adult participants, while assent and guardian consent were obtained for minors. Healthy control participants were selected from individuals referred for non-neurological complaints and found to have normal MRI findings.

Inclusion and exclusion criteria

The inclusion criteria comprised children and adults of either sex with a clinical diagnosis of unilateral TLE, confirmed by clinical evaluation and EEG findings consistent with TLE. The presence of structural abnormalities on conventional MRI was not required for inclusion. Patients with hippocampal sclerosis, hippocampal atrophy, or other temporal lobe structural changes on MRI were included when present, and patients with clinically and EEG-confirmed TLE but normal MRI findings were also eligible, in keeping with the objective of comparing conventional MRI with DTI in detecting white matter alterations.

Exclusion criteria included patients with intra-axial structural abnormalities outside the temporal lobe, as such findings could confound diffusion tensor parameters and lead to misinterpretation of white matter tract integrity. Patients with major psychiatric disorders were also excluded because of the known involvement of the uncinate fasciculus and limbic white matter in psychiatric pathologies, which could bias diffusion measurements. In addition, participants with contraindications to MRI, such as metallic implants, pacemakers, or severe claustrophobia, were excluded to ensure safety and scan quality.

Sample size calculation

The sample size for this comparative diagnostic accuracy study was calculated based on detecting a statistically significant difference in diffusion metrics specifically ADC and FA between TLE patients and controls, where *n *represents the sample size per group, *Zα/2* corresponds to the standard normal deviate for a two-tailed test at a 5% significance level (1.96), and* Zβ* corresponds to the desired statistical power (0.84 for 80% power). The pooled standard deviation (σ) was estimated at 0.20, and the minimum detectable difference between group means (Δ) was assumed to be 0.15 based on pilot data. Substituting these values yields approximately 14 participants per group to achieve adequate statistical power. Considering potential data loss and ensuring robustness, the total sample size was set at 32 participants, with 21 in the TLE group and 11 controls.

Imaging protocol

All participants underwent MRI using a GE Signa Pioneer 3.0T system (GE Healthcare, DV26.0 software) using a dedicated 32-channel head coil. The standard MRI epilepsy protocol included T1-weighted, T2-weighted, fluid-attenuated inversion recovery (FLAIR), and coronal oblique sequences perpendicular to the hippocampal axis for optimal visualization of mesial temporal structures [13]. For DTI acquisition, a single-shot spin-echo echo-planar imaging (EPI) sequence was employed with diffusion sensitization applied along 32 non-collinear directions. The diffusion weighting factor (b-value) was set at 1000 s/mm² [14,15]. The acquired data were reconstructed to generate FA and ADC maps using the vendor-provided software mentioned above.

Regions of interest (ROIs) were manually placed bilaterally over the hippocampus, parahippocampal gyrus, and selected white matter tracts, such as the fornix and cingulum, using color-coded FA maps co-registered to anatomical images. Mean FA and ADC values were extracted for each ROI. To minimize inter-observer variability, two experienced radiologists independently evaluated the images, and any discrepancies were resolved through consensus.

Data management and statistical analysis

Data entry and preliminary validation were performed using Microsoft Excel (Microsoft Corp., USA), and all statistical analyses were conducted using IBM SPSS Statistics, Version 26.0 (released 2018, IBM Corp., Armonk, NY). Descriptive statistics were expressed as mean ± standard deviation for continuous variables and as frequencies and percentages for categorical variables. The normality of quantitative data was tested using the Shapiro-Wilk test. For normally distributed data, independent sample t-tests were used to compare mean FA and ADC values between patients and controls. Non-parametric tests, such as the Mann-Whitney U test, were applied for skewed distributions. Categorical data, including demographic characteristics and qualitative imaging findings, were analyzed using the chi-square test or Fisher’s exact test as appropriate. A p-value less than 0.05 was considered statistically significant. Association analysis was performed between FA/ADC values and clinical parameters such as duration of epilepsy and seizure frequency. 

Operational definitions

MRI was defined as a non-invasive imaging modality employing magnetic fields and radiofrequency pulses to generate high-resolution anatomical images of body tissues. DTI was defined as an advanced MRI technique that quantifies the directionality and magnitude of water diffusion in brain tissue, providing insight into white matter microstructure. DWI was characterized as an imaging sequence that reflects the random motion of water molecules within tissues, aiding in the assessment of microstructural integrity. ADC represented a quantitative measure of water diffusivity, while FA quantified the degree of directionally constrained diffusion, indicative of axonal organization and integrity.

Ethical considerations

The study protocol received approval from the Institutional Ethics Committee of Sree Balaji Medical College and Hospital (002/SBMC/IHEC/2023/1968). All procedures adhered to the ethical principles outlined in the Declaration of Helsinki. Written informed consent was obtained from all participants or legal guardians in the case of minors. Participants were assured of confidentiality, and all identifying information was anonymized. They were informed about the study objectives, potential risks, and their right to withdraw at any stage without affecting clinical care. The imaging protocol was entirely non-invasive, with no contrast administration involved, and every effort was made to minimize discomfort during scanning.

## Results

The mean ADC values were consistently higher in patients with TLE compared with healthy controls, indicating altered microstructural integrity of affected neural tracts. Specifically, the mean ADC of the ipsilateral hippocampus was 0.78 ± 0.087 in cases versus 0.61 ± 0.04 in controls (t = 2.04, p = 0.04), while the contralateral hippocampus showed values of 0.88 ± 0.05 versus 0.72 ± 0.03 (t = 2.01, p = 0.04). Similarly, the ipsilateral fornix demonstrated elevated ADC (0.91 ± 0.02 vs. 0.79 ± 0.04; t = 2.17, p = 0.05), suggesting disruption of axonal coherence and water diffusivity. The contralateral posterior limb of the internal capsule exhibited the most significant difference (0.82 ± 0.04 in cases vs. 0.74 ± 0.06 in controls; t = 2.92, p = 0.006), reflecting possible contralateral tract involvement due to chronic epileptic propagation. By contrast, non-significant differences were noted in the contralateral fornix (p = 0.86), contralateral anterior limb (p = 0.134), and ipsilateral posterior limb (p = 0.149), implying relative preservation of microstructural integrity in these regions (Table 1).

**Table 1:** Comparison of ADC values between cases and controls of hippocampus and internal capsule. Independent samples t-test. *p-value <0.05 is statistically significant. ADC: apparent diffusion coefficient

Site	Cases: mean (SD)	Controls: mean (SD)	t-value	p-value
ADC of the ipsilateral hippocampus	0.78 (0.087)	0.61 (0.04)	2.04	0.04*
ADC of the contralateral hippocampus	0.88 (0.05)	0.72 (0.03)	2.01	0.04*
ADC of the ipsilateral fornix	0.91 (0.02)	0.79 (0.04)	2.17	0.05*
ADC of the contralateral fornix	0.81 (0.07)	0.82 (0.06)	0.18	0.86
ADC of the ipsilateral anterior limb of the internal capsule	0.81 (0.30)	0.74 (0.15)	2.02	0.05*
ADC of the contralateral anterior limb of the internal capsule	0.79 (0.12)	0.80 (0.24)	1.52	0.134
ADC of the ipsilateral posterior limb of the internal capsule	0.84 (0.40)	0.83 (0.21)	1.46	0.149
ADC of the contralateral posterior limb of the internal capsule	0.82 (0.04)	0.74 (0.06)	2.92	0.006*

FA values showed distinct differences between patients with TLE and healthy controls, indicating variations in white matter integrity. The mean FA of the ipsilateral hippocampus in cases was 0.34 ± 0.02, significantly higher than 0.20 ± 0.03 in controls (t = 2.12, p = 0.05), while the contralateral hippocampus also demonstrated increased FA in patients (0.36 ± 0.04) compared to controls (0.24 ± 0.03, t = 2.08, p = 0.05). This rise in FA may reflect structural reorganization or gliotic processes secondary to chronic seizure activity. In the fornix, the ipsilateral side showed a mean FA of 0.42 ± 0.02 versus 0.41 ± 0.03 in controls, which was not statistically significant (p = 0.38). However, the contralateral fornix exhibited a significant FA elevation (0.43 ± 0.04 vs. 0.39 ± 0.02, p = 0.05), indicating possible compensatory white matter changes.

The anterior limb of the internal capsule (ALIC) demonstrated a highly significant difference on the ipsilateral side, with a mean FA of 0.39 ± 0.013 in cases compared to 0.23 ± 0.013 in controls (t = 3.82, p < 0.001), suggesting disruption of frontotemporal connectivity pathways often affected in TLE. On the contralateral ALIC, FA values were lower in controls (0.45 ± 0.015 vs. 0.51 ± 0.06, p = 0.05), showing potential microstructural asymmetry linked to chronic epileptic discharge propagation. By contrast, the posterior limb of the internal capsule (PLIC) showed no significant difference on the ipsilateral side (0.51 ± 0.02 vs. 0.45 ± 0.08, p = 0.786), but a notable increase in the contralateral PLIC was observed in cases (0.54 ± 0.06 vs. 0.42 ± 0.09, t = 2.30, p = 0.05) (Table 2).

**Table 2:** Comparison of FA values between cases and controls of hippocampus and internal capsule. Independent samples t-test. *p-value <0.05 is statistically significant. FA: fractional anisotropy

Site	Cases: mean (SD)	Controls: mean (SD)	t-value	p-value
FA of the ipsilateral hippocampus	0.34 (0.02)	0.20 (0.03)	2.12	0.05*
FA of the contralateral hippocampus	0.36 (0.04)	0.24 (0.03)	2.08	0.05*
FA of the ipsilateral fornix	0.42 (0.02)	0.41 (0.03)	0.89	0.38
FA of the contralateral fornix	0.43 (0.04)	0.39 (0.02)	2.10	0.05*
FA of the ipsilateral anterior limb of the internal capsule	0.39 (0.013)	0.23 (0.013)	3.82	<0.001*
FA of the contralateral anterior limb of the internal capsule	0.45 (0.015)	0.51 (0.06)	2.01	0.05*
FA of the ipsilateral posterior limb of the internal capsule	0.51 (0.02)	0.45 (0.08)	0.82	0.786
FA of the contralateral posterior limb of the internal capsule	0.54 (0.06)	0.42 (0.09)	2.30	0.05*

In the genu of the corpus callosum, the mean FA was 0.70 ± 0.24 in cases and 0.67 ± 0.11 in controls (p = 0.19), while the ADC was 0.71 ± 0.13 and 0.69 ± 0.13, respectively (p = 0.855). For the body of the corpus callosum, FA values were 0.50 ± 0.16 in cases and 0.53 ± 0.42 in controls (p = 0.05), and ADC values were 0.83 ± 0.71 versus 0.75 ± 0.24 (p = 0.05). The splenium of the corpus callosum showed FA values of 0.76 ± 0.23 in cases and 0.61 ± 0.14 in controls (p = 0.25), and ADC values of 0.74 ± 0.72 and 0.65 ± 0.15 (p = 0.707). The ipsilateral thalamus demonstrated significantly lower FA (0.32 ± 0.06) and lower ADC (0.78 ± 0.08) compared to controls (FA = 0.52 ± 0.02; ADC = 0.92 ± 0.03; both p < 0.001). Similarly, the contralateral thalamus exhibited marked FA reduction (0.36 ± 0.05 vs. 0.73 ± 0.03; p < 0.001), although ADC values were comparable (0.72 ± 0.07 vs. 0.71 ± 0.05; p = 0.261). The inferior fronto-occipital fasciculus (IFO) also showed significant differences, with lower FA in cases (ipsilateral: 0.50 ± 0.01; contralateral: 0.55 ± 0.09) compared to controls (0.62 ± 0.04 and 0.66 ± 0.02; both p < 0.001) and higher ADC values in cases (ipsilateral: 0.79 ± 0.06; contralateral: 0.84 ± 0.03) relative to controls (0.74 ± 0.01 and 0.69 ± 0.03; both p < 0.001) (Table 3).

**Table 3:** FA and ADC values of the corpus callosum, thalamus, and inferior fronto-occipital fasciculus Independent samples t-test. *p-value <0.05 is statistically significant. ADC: apparent diffusion coefficient, FA: fractional anisotropy

Site/tract	Cases: mean (SD)	Controls: mean (SD)	t-value	P-value
FA of genu of the corpus callosum	0.70 (0.24)	0.67 (0.11)	1.34	0.19
ADC of the genu of the corpus callosum	0.71 (0.13)	0.69 (0.13)	0.19	0.855
FA of the body of the corpus callosum	0.50 (0.16)	0.53 (0.42)	2.03	0.05*
ADC of the body of the corpus callosum	0.83 (0.71)	0.75 (0.24)	2.12	0.05*
FA of the splenium of the corpus callosum	0.76 (0.23)	0.61 (0.14)	1.18	0.25
ADC of the splenium of the corpus callosum	0.74 (0.72)	0.65 (0.15)	0.38	0.707
FA of the ipsilateral thalamus	0.32 (0.06)	0.52 (0.02)	6.12	<0.001*
FA of the contralateral thalamus	0.36 (0.05)	0.73 (0.03)	7.24	<0.001*
ADC of the ipsilateral thalamus	0.78 (0.08)	0.92 (0.03)	4.97	<0.001*
ADC of the contralateral thalamus	0.72 (0.07)	0.71 (0.05)	1.15	0.261
FA of the ipsilateral IFO	0.50 (0.01)	0.62 (0.04)	5.42	<0.001*
FA of the contralateral IFO	0.55 (0.09)	0.66 (0.02)	4.91	<0.001*
ADC of the ipsilateral IFO	0.79 (0.06)	0.74 (0.01)	5.36	<0.001*
ADC of the contralateral IFO	0.84 (0.03)	0.69 (0.03)	5.77	<0.001*

In this study, FA values were significantly lower in cases compared to controls in several key regions. The ipsilateral anterior cingulum showed a mean FA of 0.30 (SD 0.07) in cases versus 0.44 (SD 0.06) in controls (p < 0.001), while the contralateral anterior cingulum had a mean FA of 0.35 (SD 0.08) in cases versus 0.44 (SD 0.06) in controls (p = 0.05). Similarly, the ipsilateral posterior cingulum demonstrated a mean FA of 0.35 (SD 0.07) in cases compared to 0.48 (SD 0.06) in controls (p < 0.001), and the contralateral posterior cingulum had 0.38 (SD 0.08) versus 0.47 (SD 0.06) (p = 0.05). For the parahippocampal gyrus, FA values were 0.35 (SD 0.08) ipsilaterally and 0.37 (SD 0.09) contralaterally in cases, compared to 0.41 (SD 0.14) in controls for both regions (p = 0.05 each). Among ADC values, only the ipsilateral posterior cingulum (0.75 ± 0.10 vs. 0.61 ± 0.14, p = 0.05) and contralateral parahippocampal gyrus (0.78 ± 0.13 vs. 0.74 ± 0.13, p = 0.05) showed significant differences. These findings indicate notable microstructural alterations in specific white matter tracts, primarily reflected by reduced FA in cases (Table 4).

**Table 4:** FA and ADC values of the anterior, posterior cingulum, and parahippocampal gyrus Independent samples t-test. *p-value <0.05 is statistically significant. ADC: apparent diffusion coefficient, FA: fractional anisotropy

Site/region	Cases: mean (SD)	Controls: mean (SD)	t-value	P-value
FA of the ipsilateral anterior cingulum	0.30 (0.07)	0.44 (0.06)	-5.23	<0.001*
FA of the contralateral anterior cingulum	0.35 (0.08)	0.44 (0.06)	-2.03	0.05*
ADC of the ipsilateral anterior cingulum	0.77 (0.10)	0.73 (0.14)	1.12	0.76
ADC of the contralateral anterior cingulum	0.76 (0.14)	0.73 (0.14)	0.64	0.54
FA of the ipsilateral posterior cingulum	0.35 (0.07)	0.48 (0.06)	-4.82	<0.001*
FA of the contralateral posterior cingulum	0.38 (0.08)	0.47 (0.06)	-2.01	0.05*
ADC of the ipsilateral posterior cingulum	0.75 (0.10)	0.61 (0.14)	2.05	0.05*
ADC of the contralateral posterior cingulum	0.70 (0.14)	0.61 (0.14)	1.37	0.67
FA of the ipsilateral parahippocampal gyrus	0.35 (0.08)	0.41 (0.14)	2.80	0.03*
FA of the contralateral parahippocampal gyrus	0.37 (0.09)	0.41 (0.14)	2.02	0.04*
ADC of the ipsilateral parahippocampal gyrus	0.80 (0.11)	0.74 (0.13)	1.57	0.43
ADC of the contralateral parahippocampal gyrus	0.78 (0.13)	0.74 (0.13)	2.00	0.01*

## Discussion

Epilepsy is a prevalent neurological disorder in which MRI plays a crucial role in diagnosis, treatment planning, and management. Despite its diagnostic value, conventional MRI fails to detect structural abnormalities in approximately 20-30% of patients with focal epilepsy, indicating its limited sensitivity [1]. Although technological advances, such as higher magnetic field strengths, improved coils, and refined imaging sequences, have enhanced spatial resolution, they have not substantially improved the diagnostic yield of conventional imaging [2]. TLE, the most common form of focal epilepsy, presents particular diagnostic and therapeutic challenges. It is frequently associated with MTS, hippocampal atrophy, and neuronal loss, often rendering patients resistant to medical therapy. These cases typically require surgical interventions such as anterior temporal lobectomy, which depend on precise imaging for identifying the epileptogenic focus and planning the resection [3].

MRI remains the cornerstone in evaluating epilepsy due to its superior soft tissue contrast and ability to visualize structural abnormalities. High-resolution T1- and T2-weighted sequences aid in detecting MTS, hippocampal atrophy, cortical dysplasia, and other epileptogenic lesions. Quantitative tools like hippocampal volumetry and T2 relaxometry have been employed to identify subtle changes not visible on qualitative scans [4]. However, a considerable proportion of TLE patients still exhibit normal MRI findings, highlighting the need for advanced imaging modalities that can provide additional insights into the brain’s microstructure. DTI has emerged as a valuable technique in this context. By quantifying the directional movement of water molecules, DTI can evaluate the integrity of white matter tracts. It provides metrics such as FA, which reflects axonal density and myelination, and ADC, which measures the magnitude of water diffusion [5]. These parameters help in detecting microscopic alterations invisible on standard MRI, offering a more sensitive assessment of tissue integrity and connectivity.

In TLE, DTI has shown that pathological changes extend beyond the temporal lobe, revealing that epilepsy is not merely a focal condition but a network disorder. Conventional MRI may demonstrate unilateral hippocampal sclerosis, yet DTI often reveals bilateral abnormalities involving distant white matter tracts such as the cingulum, fornix, corpus callosum, and thalamic connections. Such findings provide valuable insight into the widespread microstructural disruptions that underlie seizure propagation [6]. Despite its potential, DTI is not without limitations-it is sensitive to motion artifacts, magnetic field inhomogeneities, and requires careful interpretation since diffusion changes can reflect diverse pathological processes, including edema, gliosis, and demyelination [7]. Nonetheless, advancements in acquisition and post-processing techniques have improved the reliability of DTI, making it a robust complementary tool to conventional MRI.

In the present study, DTI parameters (FA and ADC) were analyzed across multiple brain regions in patients with TLE to evaluate microstructural alterations and compare them with healthy controls. The case group comprised 21 patients (nine males and 12 females), while the control group included 11 subjects (four males and seven females), showing a slight female predominance similar to prior studies by Lui YW et al. [9] and Alizadeh M et al. [10]. This matched gender distribution minimizes potential sex-related biases in diffusion analysis, supporting the validity of observed differences between groups.

Significant diffusion changes were detected in several key regions. In the hippocampus and fornix, ADC values were markedly elevated in TLE patients, suggesting microstructural disorganization. Specifically, the ipsilateral hippocampus exhibited an ADC of 0.78 ± 0.087 versus 0.61 ± 0.04 in controls (p < 0.05), and the contralateral hippocampus showed 0.88 ± 0.05 versus 0.72 ± 0.03 (p < 0.05). The ipsilateral fornix also demonstrated increased ADC (0.91 ± 0.02 vs. 0.79 ± 0.04; p < 0.05), while the contralateral fornix did not show a significant change (0.86, NS). These findings mirror those of Assaf et al. [11] and Huisman TA et al. [12], who reported increased diffusivity in the hippocampus and fornix ipsilateral to the seizure focus. The higher ADC suggests axonal disruption and gliosis in regions directly affected by epileptic activity, reflecting impaired structural integrity and water diffusion along axonal tracts.

Within the internal capsule, diffusion alterations were also evident. ADC was significantly increased in the contralateral posterior limb (0.82 ± 0.04 vs. 0.74 ± 0.06; p = 0.006), and the ipsilateral anterior limb showed a borderline rise (0.81 ± 0.3 vs. 0.74 ± 0.15; p ≤ 0.05). Rodríguez-Cruces R and Concha L [13] similarly documented increased ADC and decreased FA in these tracts, suggesting that TLE pathology extends beyond temporal structures to involve projection fibers related to motor and sensory pathways. These findings reinforce the network concept of epilepsy, demonstrating that microstructural damage transcends the primary epileptogenic zone. FA analysis of the hippocampus and fornix revealed significant reductions, consistent with compromised white matter integrity. The ipsilateral hippocampus showed FA of 0.34 ± 0.02 compared to 0.20 ± 0.03 in controls (p < 0.05), and the contralateral hippocampus 0.36 ± 0.04 versus 0.24 ± 0.03 (p < 0.05). Although the ipsilateral fornix did not differ significantly (0.42 ± 0.02 vs. 0.41 ± 0.03; p = 0.380), the contralateral fornix displayed a reduction (0.43 ± 0.04 vs. 0.39 ± 0.02; p < 0.05). These patterns correspond with findings from Knake S et al. [14] and Alexander RP et al. [15], who observed bilateral FA decreases in mesial TLE, reflecting widespread axonal compromise. The asymmetric involvement may represent both primary hippocampal degeneration and secondary trans-synaptic effects.

Analysis of the internal capsule’s FA revealed decreased integrity in the ipsilateral anterior limb (0.39 ± 0.013 vs. 0.23 ± 0.013; p < 0.001) and contralateral posterior limb (0.54 ± 0.06 vs. 0.42 ± 0.09; p < 0.05), consistent with prior DTI studies indicating bilateral motor projection tract disruption [13,16]. These reductions in FA may correlate with chronic seizure activity and disease duration, potentially impacting motor and cognitive performance. No significant group difference was observed in the genu; the body showed borderline differences, and the splenium was non-significant on conventional thresholds. These findings do not support a large genu effect in our cohort and suggest that interhemispheric involvement, when present, may preferentially affect non-genu segments.. These findings mirror those of Rodríguez-Cruces R et al. [13] and Stasenko A et al. [17], who linked callosal FA decreases to interhemispheric disconnection and chronic disease effects. The pattern supports bilateral callosal involvement, reflecting impaired interhemispheric transfer and network disruption extending beyond temporal regions.

In the thalamus, both FA and ADC demonstrated significant alterations. The ipsilateral thalamus showed reduced FA (0.32 ± 0.06 vs. 0.52 ± 0.02; p < 0.001) and lower ADC (0.78 ± 0.08 vs. 0.92 ± 0.03; p < 0.001), while the contralateral thalamus had reduced FA (0.36 ± 0.05 vs. 0.73 ± 0.03; p < 0.001) without ADC change (p = 0.261). These findings corroborate the results of Kimiwada et al. [18] and Thivard L et al. [19], who described thalamic diffusion changes linked to secondary seizure generalization. The bilateral FA reduction and ipsilateral ADC decrease suggest disrupted thalamo-cortical connectivity and neuronal loss, contributing to seizure propagation mechanisms. DTI findings in the IFO also indicated extensive network involvement. Both ipsilateral (FA 0.50 vs. 0.62) and contralateral (FA 0.55 vs. 0.66) sides showed significant reductions (p < 0.001), while ADC increased (ipsilateral 0.79 vs. 0.74; contralateral 0.84 vs. 0.69; p < 0.001). Campos BM et al. [20] and Lemkaddem A et al. [21] similarly reported FA decline and diffusivity increase in the IFO, correlating with cognitive impairment and seizure burden. These findings confirm the role of associative white matter tracts in the pathophysiology of TLE.

In the cingulum, anterior segment FA was significantly reduced on both sides (ipsilateral 0.30 ± 0.07 vs. 0.44 ± 0.06; p < 0.001; contralateral 0.35 ± 0.08 vs. 0.44 ± 0.06; p < 0.05) with no significant ADC change, indicating axonal disruption without major extracellular alteration [22,23]. The posterior cingulum also showed decreased FA (ipsilateral 0.35 ± 0.07 vs. 0.48 ± 0.06; p < 0.001; contralateral 0.38 ± 0.08 vs. 0.47 ± 0.06; p < 0.05) and elevated ipsilateral ADC (0.75 ± 0.10 vs. 0.61 ± 0.14; p < 0.05), suggesting focal demyelination. Finally, in the parahippocampal gyrus, FA was lower bilaterally (ipsilateral 0.35 vs. 0.41; contralateral 0.37 vs. 0.41; p < 0.05), and contralateral ADC was elevated (0.78 vs. 0.74; p < 0.05), in agreement with Rodríguez-Cruces R and Concha L [13]. Collectively, these results demonstrate that DTI detects microstructural abnormalities in both temporal and extratemporal regions in TLE. Reduced FA across multiple tracts indicates widespread axonal degeneration and demyelination, while increased ADC in selected regions reflects extracellular diffusion changes secondary to neuronal loss. The findings support the concept of TLE as a network disorder involving limbic, callosal, thalamic, and associative pathways.

This study, however, is not without limitations. The relatively small sample size may limit generalizability and statistical power. Manual region-of-interest placement could introduce observer bias, and the cross-sectional design precludes causal inference regarding disease progression. Additionally, potential confounders such as duration of epilepsy, medication effects, and seizure frequency were not fully controlled. Future studies with larger cohorts, automated tract-based spatial statistics (TBSS), and longitudinal follow-up could enhance accuracy and elucidate the temporal evolution of white matter changes.

The implications of these findings are substantial. DTI can serve as a complementary tool to conventional MRI, particularly for patients with MRI-negative epilepsy, by revealing subtle microstructural abnormalities. Incorporating DTI into pre-surgical evaluations could improve localization of epileptogenic zones, optimize surgical planning, and potentially predict postoperative outcomes. Moreover, by mapping network-level changes, DTI may help in understanding cognitive and behavioral deficits associated with chronic epilepsy, paving the way for more targeted therapeutic interventions and precision-based neuroimaging approaches in epilepsy management. Future research should explore longitudinal DTI changes to understand disease progression, investigate correlations between DTI metrics and cognitive outcomes, and validate these findings in larger multi-center cohorts. Integration of DTI with advanced techniques like diffusion kurtosis imaging and neurite orientation dispersion density imaging may provide even greater sensitivity for detecting microstructural changes.

## Conclusions

The study demonstrated significant microstructural white matter alterations in the anterior and posterior cingulum and parahippocampal gyrus among cases compared to controls, reflected by consistently reduced fractional anisotropy and variably increased apparent diffusion coefficient values. These findings suggest compromised fiber integrity and disrupted connectivity in key limbic and associative pathways, underscoring the potential of DTI parameters, particularly FA, as sensitive markers for detecting subtle white matter abnormalities not visible on conventional MRI.
